# Integrating autonomously navigating assistance systems into the clinic: guiding principles and the ANTS-OR approach

**DOI:** 10.1007/s11548-020-02137-8

**Published:** 2020-04-02

**Authors:** Lukas Bernhard, Daniel Ostler, Hubertus Feußner, Dirk Wilhelm

**Affiliations:** 1 Research Group MITI, Klinikum rechts der Isar der Technischen Universität München, Munich, Germany; 2 Department of Surgery, Klinikum rechts der Isar der Technischen Universität München, Munich, Germany

**Keywords:** Self-navigating systems, Clinical assistance systems, Scheduling, Clinical workflow optimization

## Abstract

**Purpose:**

Autonomously self-navigating clinical assistance systems (ASCAS) seem highly promising for improving clinical workflows. There is great potential for easing staff workload and improving overall efficiency by reducing monotonous and physically demanding tasks. However, a seamless integration of such systems into complex human-supervised clinical workflows is challenging. As of yet, guiding principles and specific approaches for solving this problem are lacking.

**Methods:**

We propose to treat ASCAS orchestration as a scheduling problem. However, underlying objectives and constraints for this scheduling problem differ considerably from those found in other domains (e.g., manufacturing, logistics). We analyze the clinical environment to deduce unique needs and conclude that existing scheduling approaches are not sufficient to overcome these challenges.

**Results:**

We present four guiding principles, namely *human precedence, command structure, emergency context* and *immediacy,* that govern the integration of self-navigating assistance systems into clinical workflows. Based on these results, we propose our approach, namely *Auto*-*Navigation Task Scheduling for Operating Rooms* (ANTS-OR), for solving the ASCAS orchestration problem in a surgical application scenario, employing a score-based scheduling strategy.

**Conclusion:**

The proposed approach is a first step toward addressing the ASCAS orchestration problem for the OR wing. We are currently advancing and validating our concept using a simulation environment and aim at realizing a dynamic end-to-end ASCAS orchestration platform in the future.

## Purpose

Mobile self-navigating robotic technology has successfully been applied to various domains, including logistics [1], housekeeping [2], agriculture [3], exploration [4], customer service [5], maintenance in hazardous environments [6] and delivery of goods [7]. Due to their high level of autonomy—often combined with versatile interfaces to the environment—robotic systems seem highly promising for the health-care domain, especially when dealing with pressing social problems like overaging and shortage of qualified personnel. There is great potential for easing staff workload and improving overall efficiency by reducing monotonous and physically demanding tasks.

First concepts for domestic care—e.g., *GARMI* (Franka Emika, Munich, Germany), *Twendy*-*One* [8] and *Care*-*O*-*bot* [9]—as well as clinical care—e.g., *Moxi* (Diligent Robotics, Austin, USA) and *RIBA* [10]—have been presented. Complementarily, we anticipate that *autonomously self*-*navigating clinical assistance systems* (ASCAS) will play a crucial role in making clinical workflows more efficient, safe and ergonomic. Possible applications are manifold and include simple fetching of materials, assisting in repositioning of patients, device control, documentation tasks, inventory management and more. In particular, we envision ASCAS as a central component of the fully assisted OR environment of the future. Within such an environment, robotic team members collaborate closely and continuously with their human counterparts to guarantee patient safety and improve patient outcome, while making surgical processes more robust and efficient. Prospectively, this might lead to a partial or even complete merging of robotic and human spheres of influence within the clinic. This is in stark contrast to most industrial and domestic scenarios where robotic machines are usually operating in highly optimized but delimited environments, commonly called *envelopes*, to complete subtasks of the production workflow in an unhampered manner.

Consequently, fundamental challenges must be addressed before a broad integration of ASCAS to the clinic can become a reality. We need to find means to seamlessly integrate these systems into complex clinical workflows. Guiding principles for achieving this in a safe, ethical and economic way that leaves clinicians in control of the workflow while maximizing productivity are needed.

In this short communication, we aim at providing such principles, to govern human–machine collaboration in the clinic and leverage the full potential of ASCAS regarding workflow optimization. As a further key contribution, we apply our principles to a surgical application scenario and utilize them to derive an explicit scheduling strategy named *Auto*-*Navigation Task Scheduling for Operating Rooms* (ANTS-OR) for ASCAS orchestration across multiple operating rooms.

## Methods

The ASCAS orchestration problem may be described as follows: There are *n* ASCAS deployed within a clinical unit (ward, OR wing, etc.). Each ASCAS offers a set of *tasks* that it can perform (e.g., fetching sterile material, moving patient beds, adjusting medical devices). Multiple ASCAS may offer the same type of task. The execution of a task takes a certain—in general unknown—amount of time and may require a change of location beforehand. Tasks may be assigned or canceled at any time by members of the clinical staff or by clinical information systems. The execution of a task may depend on the completion of another task, and thus underlie *precedence* relations, or depend on other preconditions. The overall goal is an optimal exploitation of the available ASCAS resources regarding one or more *objectives* (e.g., patient well-being, patient outcome, staff ergonomics, throughput, costs).

Clearly, this is closely related to *scheduling problems* that are common in many different domains including logistics, operational research, manufacturing and computer multitasking. Many variants of these problems have been described to address unique constraints or objectives associated with different contexts [11]. The ASCAS orchestration problem is characterized by its *dynamic* nature, since new tasks may be assigned at any time. Special consideration must also be given to *objectives* and *constraints* of the scheduling. As in the manufacturing or logistics domain, *time*- and *cost*-related objectives must be considered, though other factors, such as patient welfare, patient outcome and staff ergonomics, are of higher relevance. Tasks in the ASCAS orchestration problem are also subject to certain constraints, introduced by clinical command structures, emergency situations and precedence relations between tasks. Due to these unique and partly indistinct objectives and constraints, developing solutions to ASCAS orchestration based on known optimal or suboptimal scheduling algorithms is not straightforward.

## Results

Inspired by Isaac Asimov’s famous *Laws of Robotics*, we propose a set of fundamental principles that must be considered by any approach dealing with ASCAS orchestration. These principles aim at reflecting the unique requirements associated with clinical environments and describe the governing rules of human–machine collaboration in a simple and solution-independent manner. Based on these results, we propose our ANTS-OR approach for solving the ASCAS orchestration problem in a surgical application scenario, employing a score-based scheduling strategy.

### Principles of ASCAS orchestration

(*P1*) *Human Precedence: The clinical staff must remain in control of the workflow* (*P1a*) *and be aware of all autonomously performed actions and their consequences* (*P1b*). In modern clinics, processes are not only controlled by human beings but also by clinical information systems. In the future, these systems might be capable of (semi-)autonomously assigning tasks to ASCAS resources: For example, a bed transportation robot might be dispatched to transfer the next patient from ward to operating room, as soon as the end of the previous surgery has been registered in the clinical information system. With the advent of AI-driven workflow recognition technology, the influence of autonomous decision systems might increase even more: imagine a workflow recognition engine—e.g., tracking the progress of a surgical intervention—assigning ASCAS tasks on the fly to offer automated context-dependent support for the surgical team. While these new technologies seem promising for improving clinical efficiency, it is vital to ensure that human staff remains in control of the process at any time.

(*P2*) *Command Structure: Decisions made by senior team members supersede decisions made by subordinates.* Clinical workflows and decision-making processes are grounded on clear hierarchies that allow for efficient collaboration, especially in frequently occurring critical and time-sensitive situations. These command structures must be conserved when dealing with ASCAS orchestration, e.g., by attaching a higher priority to tasks assigned by senior staff members than to tasks assigned by subordinates.

(*P3*) *Emergency Context: Measures dealing with emergency situations must be executed with maximum priority.* Time-sensitive situations, e.g., when dealing with emergencies or adverse events, are occurring frequently in clinical units. These situations demand immediate measures to avoid or minimize severe consequences for the patient. Thus, tasks that are assigned in an emergency context or are inherently emergency related must be executed with minimal delay. Other upcoming tasks that are not emergency related must stand back until the critical situation has been resolved.

(*P4*) *Immediacy: Timespan between task assignment and start of execution must be minimized.* Even in a non-emergency context, immediate execution of newly assigned tasks is desired in clinical workflows. In contrast to, e.g., the manufacturing domain, tasks are normally not associated with a precise due date or deadline but are supposed to be processed as soon as possible after being released (assigned). One can define the current *idle time* for each released task as difference between the *current time* and the task’s *release time*. We propose that a task should gain priority in ASCAS scheduling with increasing idle time.

### ANTS-OR approach for ASCAS orchestration in workflow-assisted surgical interventions

ASCAS systems are a promising technology for supporting surgical teams during interventions and thus improving the overall workflow in the OR wing. We envision the following scenario for the scheduling approach presented in this section: Multiple operating rooms are running in parallel within an OR wing, each one with its own schedule of surgeries that are being processed by surgical teams. Some of these operating rooms may be equipped with workflow assistance technology that is able to track the surgical workflow and derive context-dependent supportive actions. The OR tract features a set of ASCAS with different abilities (e.g., fetching of materials or control of medical devices). Tasks may be assigned or canceled by the surgical staff or by the workflow assistance engine at any time.

We make the following simplifying assumptions: Firstly, tasks are non-preemptive. Secondly, there are no precedence relations or other preconditions. Thus, assigning a task implies that it is execution ready. Thirdly, traveling durations are known for all relevant ASCAS routes.

**Fig. 1:**
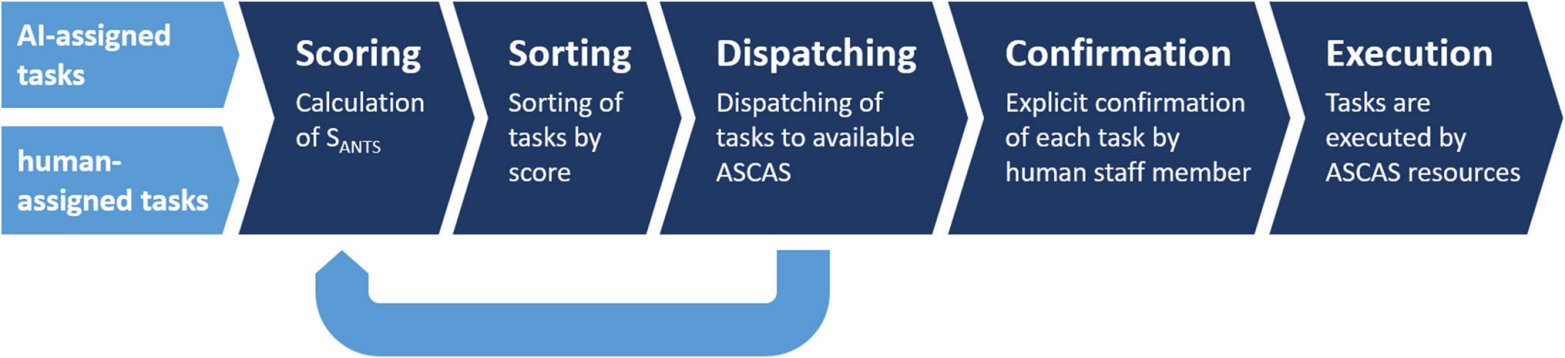
ASCAS orchestration strategy for workflow-assisted surgical interventions

**Fig. 2:**
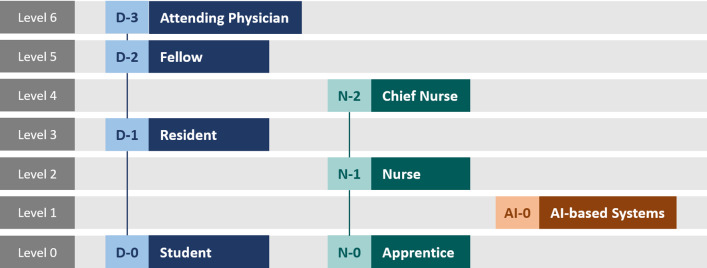
Command level hierarchy for ASCAS scheduling
$$ LEVEL_{E} $$
Emergency level (Fig. 3),

Figure 1 summarizes all major steps of the proposed scheduling strategy. Incoming tasks—either assigned by the workflow assistance engine or by human staff members—are added to a global task list maintained by the scheduler. Prioritization is done by calculating the multiparameter score $S_{ANTS}$ for each task in the global list:

$$ \begin{aligned}S_{ANTS} \left( t \right) &= w_{C} *LEVEL_{C}   +  w_{E} *LEVEL_{E}   \\& \quad +  w_{I} *f_{I} \left( {t - t_{release} } \right) \end{aligned}$$

$$ S_{ANTS} $$
ANTS-OR score,
$$ LEVEL_{C} $$
Command level (Fig. 2),

**Fig. 3:**
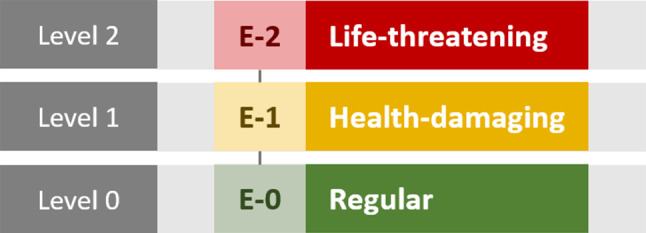
Emergency level hierarchy for ASCAS scheduling
$$ f_{I} $$
Idle time function,
$$ t_{release} $$
Task release time,
$$ w_{C} , w_{E} , w_{I} $$
Balancing weights.

To address ASCAS orchestration principles (P2) and (P3), we introduced the *command and emergency levels* depicted in Figs. 2 and 3. These levels are incorporated into the score as integer parameters $LEVEL_{C}$ (command level) and $LEVEL_{E}$ (emergency level). This ensures that task priority increases for higher command and/or emergency levels.

As shown in Fig. 2, AI-based systems (such as workflow assistance engines) have been included in the command hierarchy. Since (P1a) requires that humans must remain in control of the workflow at any time, we placed AI-based systems on a dedicated command level below human staff members (students and apprentices excepted). Thus, human-originated tasks are favored over AI-originated tasks and are expected to have shorter idle times. However, this does not mean that erroneous AI-originated tasks—e.g., caused by insufficient workflow recognition—can be replaced or corrected by dedicated human-originated task assignments this way. For that, we propose an additional safety routine during which an AI-task must explicitly be confirmed by an authorized staff member. This simultaneously enforces principle (P1b), since staff members are being made aware of all AI-based actions.

Since (P4) requires idle times to be as short as possible, we incorporated the time-dependent function $f_{I}$ into the score. By definition, this function yields higher values for longer idle times ($t - t_{release}$) and thus increases the task’s score over time. This ensures that even tasks with low emergency or command levels are being executed eventually and not constantly being blocked by higher-level tasks. The optimal choice for $f_{I}$ is still subject of ongoing research, though we plan to benchmark different polynomial and exponential behaviors.

The factors $w_{C}$, $w_{E}$ and $w_{I}$ are weights for adjusting the influence of the respective parameters and thus of different ASCAS orchestration principles. As of yet, the optimal values for these weights are free design parameters that still need to be determined in the future, based on firsthand experience or simulation.

After scoring, tasks in the global list are sorted by score. Starting from the top of the global list, the scheduler now tries to match tasks from the global list with ASCAS systems based on individual capabilities and current occupation. In case that more than one ASCAS is available and capable of executing the task, the candidate with the shortest traveling duration to task location is chosen in order to improve overall throughput.

The following example illustrates the concept described above for a simple scenario: Let’s suppose four operating rooms are being in use within an OR wing. The human staff members are supported by a single multifunctional ASCAS that is able to fetch supplies as well as operate medical devices. In OR 1, a laparoscopic cholecystectomy (gallbladder removal) is conducted, while the surgical workflow is tracked and assisted by an AI algorithm. Just now, this algorithm has assigned a new task *T*_1_ with the goal of modifying the position of the OR table to prepare for wound closure. In OR 2, the surgical team is currently facing a severe bleeding during a partial hepatectomy. An ASCAS task *T*_2_ is assigned by the attending surgeon with the goal of fetching new blood bags. OR 3 is currently being prepared for an upcoming surgery by the nursing team. The ASCAS is ordered to reset the surgical devices (task *T*_3_). In OR 4, a port implantation is being performed by a surgical resident under the supervision of an attending surgeon. The resident orders the ASCAS to fetch suture material (task *T*_4_).

Provided that all tasks have been assigned at the same time, Table 1 summarizes the $S_{ANTS}$ score for each task and the resulting processing order (rank). Idle times have been omitted, since—due to the simultaneous assignment—values are identical for any given future point in time. The weights $w_{C}$ and $w_{E}$ have been chosen such that command level and emergency level contribute equally to the score ($w_{C} = 1$; $w_{E} = 3$).

**Table 1:** Score values and resulting ranks for tasks *T*_1_ to *T*_4_ according to the introduced exemplary scenario

	$LEVEL_{C}$	$LEVEL_{E}$	$S_{ANTS}$	Rank
*T*_1_	1	0	1	4
*T*_2_	6	2	12	1
*T*_3_	2	0	2	3
*T*_4_	3	0	3	2

Thus, the highest score value is obtained for task *T*_2_, which is reasonable, since it is originating from a high-ranking team member and dealing with an emergency situation. Other tasks have to stand back until the execution of *T*_2_ is finished.

## Conclusion

The proposed scheduling approach ANTS-OR is a first step toward addressing the ASCAS orchestration problem for the OR wing. We are currently evaluating our concept using a simulation environment, where we benchmark different assisted and non-assisted scenarios to fine-tune and validate the algorithm. Besides improving our score-based approach, we aim at exploring and adapting known optimal and suboptimal scheduling algorithms to realize a dynamic end-to-end ASCAS orchestration platform in the future.
